# The effect of body mass index, lower extremity performance, and use of a private car on incident life-space restriction: a two-year follow-up study

**DOI:** 10.1186/s12877-018-0956-3

**Published:** 2018-11-08

**Authors:** Taishi Tsuji, Merja Rantakokko, Erja Portegijs, Anne Viljanen, Taina Rantanen

**Affiliations:** 1 Center for Preventive Medical Sciences, Chiba University, 1-8-1 Inohana, Chuo Ward, Chiba City, Chiba, 260-8670 Japan; 2 Gerontology Research Center, Faculty of Sport and Health Sciences, University of Jyvaskyla, PO Box 35, FI-40014 Jyväskylä, Finland

**Keywords:** Mobility limitation, Obesity, Physical performance, Aging

## Abstract

**Background:**

The purpose of the study was to explore the single and combined contributions of body mass index (BMI) and lower extremity performance as modifiable physical factors, and the influence of use of a private car as an environmental factor on prevalent and incident life-space restriction in community-dwelling older people.

**Methods:**

Community-dwelling people aged 75–90 years (*n* = 823) participated in the Life-Space Mobility in Old Age (LISPE) two-year follow-up study. Participants who reported that the largest life-space area they had attained, without aid from any device or another person, was the neighborhood or less were considered to have life-space restriction. Incident life-space restriction was the endpoint of Cox’s proportional hazard model. BMI, lower extremity performance (Short Physical Performance Battery, SPPB), and use of a private car were predictors.

**Results:**

At baseline, people who had both obesity (BMI ≥30.0) and impaired lower extremity performance (SPPB 0–9) had a higher prevalence of life-space restriction (prevalence ratio 3.6, 95% confidence interval, CI, 2.0–6.3) compared to those with normal weight (BMI 23.0–24.9) and intact physical performance (SPPB 10–12). The 581 people without life-space restriction at the baseline contributed 1033 person-years during the two-year follow-up. Incident life-space restrictions were reported by 28.3% participants. A higher hazard ratio (HR) for incident life-space restriction was observed in subjects having both obesity and impaired lower extremity performance (HR 3.6, 95% CI, 1.7–7.4), impaired lower extremity performance only (HR 1.9, 95% CI 0.9–4.1), and obesity only (HR 1.8, 95% CI, 0.9–3.5) compared to those with normal weight and intact performance. Private car passengers (HR 2.0, 95% CI, 1.3–3.0) compared to car drivers had a higher risk of life-space restriction. All models were adjusted for age, sex, chronic diseases, and education.

**Conclusions:**

Older people with impaired lower extremity performance have an increased risk of incident life-space restriction especially if combined with obesity. Also, not driving a car renders older people vulnerable to life-space restriction.

## Background

The maintenance of good mobility is vital to attaining active aging, being closely linked to physical and psychological health status and quality of life [1, 2]. Life-space mobility refers to the spatial extent of the actual mobility performance, which depends on the balance between older adults’ internal physiologic capacity and the external challenges and resources encountered in their daily environment [3]. Previous cross-sectional studies have revealed that life-space mobility is positively associated with physical [4–6] and psychological health [4] and quality of life [7, 8]. Furthermore, low life-space mobility predicts future falls [9], incident activities of daily living (ADL) disability [10], a rapid decline in cognitive function [11], health care utilization [12], and premature death [13]. Few reports have investigated the factors that may induce the incidence of life-space restriction. To date, hearing difficulties [14], low executive function [15], and frailty [16] have been reported as factors causing life-space restriction.

Life-space mobility considers the size of the spatial area (bedroom, home, yard outside home, neighborhood, town, distant locations) a person purposely moves through in daily life and the frequency of travel within a specific time and needs for assistance for that travel [4]. Movement through smaller life-space areas is more likely to occur using active forms of transportation, such as walking. In turn, traveling longer distances is more dependent on using a car or other modes of passive transportation [17]. Several cross-sectional studies have suggested that driving a car is associated with a larger life-space [18–20]. Consequently, the absence of a private car, which can be driven at will, may be a crucial environmental factor that negatively affects life-space.

Body mass index (BMI) and lower extremity performance are modifiable physical characteristics. Earlier studies have indicated that low life-space mobility is more widespread among very obese (BMI ≥35.0 kg/m^2^) community-dwelling older individuals [5, 21]. The lower extremity performance of older individuals contributes substantially to the variability in life-space mobility [4–6]. Portegijs et al. [6] reported that poor balance, walking speed, and chair standing test performance were associated with reduced life-space mobility. Obesity and poor lower extremity performance may cause life-space restriction, which in turn, may result in a vicious cycle of increasing body weight and decreasing performance.

The present study aimed to explore the single and combined contributions of BMI and lower extremity performance and the use of a private car on incident life-space restriction in community-dwelling older people.

## Methods

### Study design and participants

The Life-Space Mobility in Old Age (LISPE) project, a two-year prospective cohort study, included 848 community-dwelling individuals aged 75–90 years residing in the municipalities of Jyväskylä and Muurame, Finland. The participants were recruited from a random sample drawn from the national population register. The inclusion criteria were a willingness to participate, community-dwelling in the study area, and ability to communicate. Participants were interviewed in their homes during spring 2012 and followed up by telephone one (mean 362 ± 9 days) and 2 years (mean 721 ± 8 days) after the baseline assessment. The recruitment and study methods, including nonrespondents, have been previously published [3].

**Fig. 1:**
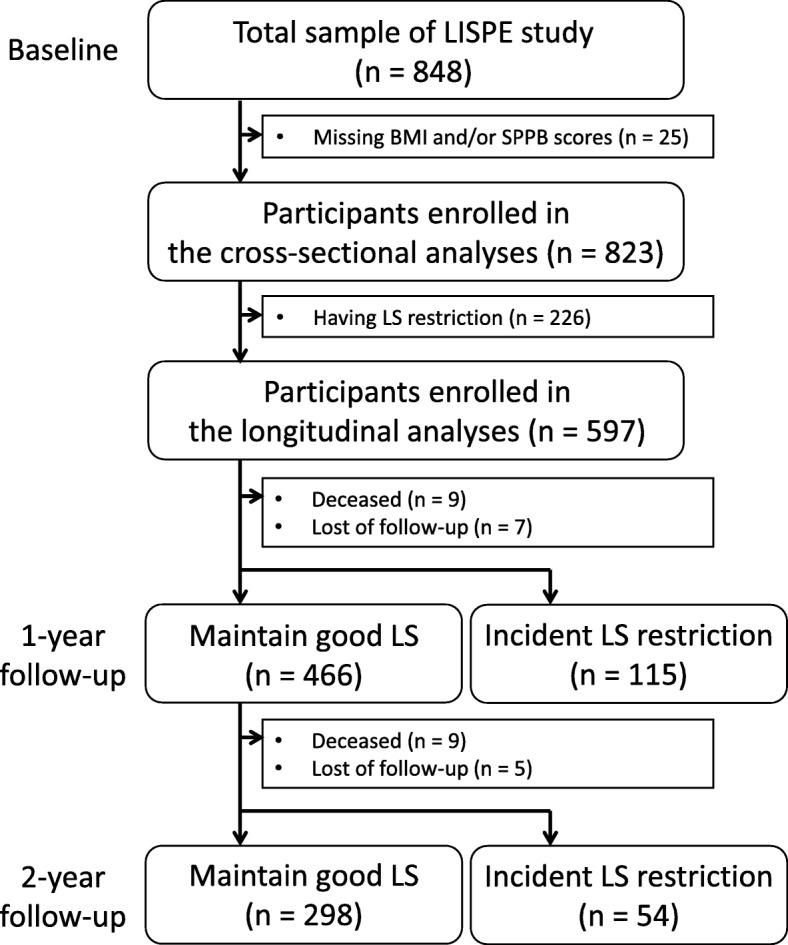
Flow of participants in the study from the Life-space mobility in old age (LISPE) project 2012–2014. LISPE: the Life-space mobility in old age study, BMI: body mass index, LS: life-space

Figure 1 shows the procedure for sample selection in our study. Of the baseline participants, those with missing data pertaining to BMI or lower extremity performance (*n* = 25) were excluded from the cross-sectional analyses leaving 823 participants. After excluding participants with life-space restriction (*n* = 226), 597 participants (262 male and 335 female) were included in the present longitudinal analyses.

### Measurements

#### Life-space mobility

Life-space mobility was assessed with the 15-item University of Alabama at Birmingham Study of Aging Life-Space Assessment [4] in a face-to-face interview at the baseline and telephone interviews at the 1st and 2nd follow-ups. Participants reported how many days a week they had attained each life-space level (0, bedroom; 1, other rooms; 2, outside home; 3, neighborhood; 4, town; 5, beyond town) during the preceding 4 weeks and whether they needed help from another person or assistive devices. When the largest life-space area attained without assistance from any device or another person was the neighborhood or less (i.e., maximal independent life-space score ≤ 3), subjects were considered to have life-space restriction. Subjects admitted to institutional care were also assigned incident life-space restriction.

#### Body mass index

Self-reported body height and weight were obtained at the baseline. BMI was calculated as weight (kg) divided by height squared (m^2^). We classified participants as obese (≥30.0 kg/m^2^) and overweight (25.0–29.9 kg/m^2^) based on World Health Organization (WHO) guidelines [22]. We categorized those participants as normal weight (23.0–24.9 kg/m^2^) and low weight (< 23.0 kg/m^2^) based on the Mini Nutritional Assessment [23]. As only seven of the participants met the WHO criteria for underweight (< 18.5 kg/m^2^) in the longitudinal analyses.

#### Lower extremity performance

We objectively evaluated lower extremity performance using the Short Physical Performance Battery (SPPB) [24] at baseline. SPPB comprises three tests assessing standing balance, walking speed over 2.44 m, and five timed chair rises. Each task was scored 0–4 points according to established age- and sex-specific cutoff points [25]. A sum score was calculated (range, 0–12). When at least two tests were completed the result was included in the analyses and being scaled to represent the range. Higher scores indicated better performance. We categorized the participants as having intact (10–12) or impaired (0–9) physical performance.

#### Use of a private car

At the baseline, participants were asked how often they drive a car or travel by car as a passenger. The response options were daily or almost daily, a few times a week, a few times a month, a few times a year, less than once a year, and never. To be categorized as a car driver, or a private car passenger, participants needed to report using a private car at least on a monthly basis. In case they reported both drivers and being a passenger, the more frequent role was selected for the categorization. If the frequency of driving and traveling as a passenger were equal, the participant was categorized as a car driver. If the participant reported using a private car less than monthly, the person was categorized as not using a private car.

#### Covariates

Information regarding age and sex was derived from the national population register. Years of education was self-reported. The number of chronic diseases was calculated from a list of 22 self-reported, physician-diagnosed chronic diseases (asthma, chronic obstructive pulmonary disease, chronic bronchitis, myocardial infarction, coronary heart disease, heart failure, hypertension, stroke, thrombosis, rheumatic arthritis, osteoarthritis, chronic back pain or problems, chronic neck pain or problems, cataract (not surgically repaired), glaucoma, macular degeneration, hearing disorders, diabetes mellitus, malignant cancer, Parkinson’s disease, Alzheimer’s disease or dementia, depression or other psychiatric disorder) and an additional open-ended question concerning any other physician-diagnosed chronic conditions [6].

### Statistical analyses

Group differences in baseline characteristics were investigated using ANOVA, chi-square, Kruskal-Wallis, and Mann-Whitney *U* tests. The main outcome of this study was incident life-space restriction. A multivariate Poisson regression analysis was conducted to confirm the cross-sectional association of each BMI and SPPB category and the use of a private car with the prevalence of life-space restriction. The results were presented as prevalence ratios (PRs) with 95% confidence intervals (CIs). Because the percentages of individuals with life-space restriction (27.5%) were > 10%, the adjusted odds ratio derived from logistic regression could no longer approximate the PR [26]. Cox’s proportional hazards models were used to test the longitudinal association of each BMI and SPPB category and the use of a private car with incident life-space restriction. Ties were handled using the Breslow method. The results were presented as hazard ratios (HRs) with 95% CIs. The follow-up period for each participant was defined from the baseline examination to the time point of the first incidence of life-space restriction, the time point of death, or the last study contact, whichever came first (i.e., 1st or 2nd follow-up) to calculate person-years. Five models were constructed in both the cross-sectional and longitudinal analyses. Model 1 and 2 independently included BMI (with normal weight as reference) and SPPB categories (with intact physical performance as reference), respectively. BMI and SPPB categories were jointly included in model 3 and covariates were added. After creating eight dummy variables by combining 4 BMI × 2 SPPB categories, these and all covariates were included in model 4 with normal weight and intact physical performance as a reference, and use of a private car was added in model 5 with car driver as a reference. SPSS Statistics 22 (IBM, Armonk, NY, USA) was used for all statistical analyses. The statistical significance was set at *P* < 0.05.

## Results

Table 1 shows participants’ characteristics according to both BMI and SPPB categories. Participants with a BMI ≥30.0 kg/m^2^ were more often women and younger than subjects with a lower BMI. The most prevalent use of a private car in participants with normal weight was driving; whereas in those with low weight, obesity, or who were overweight, being a passenger was most common. Those with impaired lower extremity performance were older and had a higher BMI, more chronic diseases, less education, and more restricted life-space area than those with intact lower extremity performance.

**Table 1:** Descriptive baseline data of participants according to BMI and lower extremity performance

Variables	Low weight (BMI < 23.0)	Normal weight (BMI 23.0–24.9)	Overweight (BMI 25.0–29.9)	Obese (BMI ≥30.0)	*P*	Intact LEP (SPPB 10–12)	Impaired LEP (SPPB 0–9)	*P*
	*n* = 156	*n* = 166	*n* = 375	*n* = 126		*n* = 523	*n* = 300	
Characteristics								
Age (year)*, M ± SD*	81.2 ± 4.3	79.9 ± 4.2	80.0 ± 4.3	79.4 ± 4.1	.003 ^a^	79.7 ± 4.2	80.8 ± 4.3	<.001 ^a^
Women, *n (%)*	100 (64.1%)	88 (53.0%)	226 (60.3%)	97 (77.0%)	<.001 ^b^	318 (60.8%)	193 (64.3%)	.315 ^b^
Height (cm), *M ± SD*	163.8 ± 9.0	166.7 ± 9.0	164.9 ± 9.0	162.4 ± 7.7	<.001 ^a^	165.0 ± 8.4	164.0 ± 9.7	.104 ^a^
Body weight (kg), *M ± SD*	56.8 ± 8.3	66.9 ± 7.4	73.7 ± 8.4	86.9 ± 11.0	<.001 ^a^	70.8 ± 11.8	71.7 ± 13.8	.317 ^a^
BMI (kg/m2), *M ± SD*	21.1 ± 1.6	24.0 ± 0.5	27.0 ± 1.4	32.9 ± 2.9	<.001 ^a^	26.0 ± 3.8	26.6 ± 4.3	.029 ^a^
Chronic diseases (n), *Med (IQR)*	4 (4)	4 (3)	4 (3)	4 (3)	.007 ^c^	4 (3)	5 (4)	<.001 ^d^
Education (year), *Med (IQR)*	9 (5)	9 (5)	8 (5)	8 (5)	.060 ^c^	9 (5)	8 (4)	<.001 ^d^
Life-space								
Maximal independent LS (score), *Med (IQR)*	4 (2)	5 (1)	4 (2)	4 (4)	.019 ^c^	5 (1)	4 (4)	<.001 ^d^
Short Physical Performance Battery								
Total (score), *Med (IQR)*	11.0 (3.0)	11.0 (3.0)	10.0 (3.0)	10.0 (3.0)	.006 ^c^	11.0 (1.0)	8.0 .0)	<.001 ^d^
Impaired (SPPB 0–9), *n (%)*	56 (35.9%)	48 (28.9%)	144 (38.4%)	52 (41.3%)	.113 ^b^	–	–	
Use of a private car, *n (%)*								
Car driver	51 (32.7%)	78 (47.0%)	160 (42.7%)	37 (29.4%)	.006 ^b^	246 (47.0%)	80 (26.7%)	<.001 ^b^
Private car passenger	77 (49.4%)	66 (39.8%)	165 (44.0%)	75 (59.5%)		216 (41.3%)	167 (55.7%)	
No use of a private car	28 (17.9%)	22 (13.3%)	50 (13.3%)	14 (11.1%)		61 (11.7%)	53 (17.7%)	

*n* = 823

*M* mean, *SD* standard deviation, *Med* median, *IQR* interquartile range, *BMI* body mass index, *LEP* lower extremity performance, *SPPB* short physical performance battery, *LS* life-space

^a^one-way analysis of variance; ^b^chi-square test; ^c^Kruskal-Wallis test; ^d^Mann-Whitney *U* test

Table 2 shows the results of multivariate Poisson regression analyses having life-space restriction at the baseline. Among those with impaired performance, all BMI groups had a significantly higher risk of restricted life-space than the group with normal weight and intact physical performance in the fully adjusted model (PR varies between 2.71 and 3.31).

**Table 2:** Association of BMI and lower extremity performance with life-space restriction (cross-sectional analysis)

	Model 1		Model 2		Model 3^a^		Model 4^a^		Model 5^a^	
	PR	95% CI	PR	95% CI	PR	95% CI	PR	95% CI	PR	95% CI
Single association										
BMI										
< 23	1.36	(0.93, 1.98)			1.03	(0.74, 1.44)				
23–25	1.00				1.00					
25–30	1.21	(0.86, 1.69)			1.01	(0.75, 1.36)				
≥ 30	1.68	(1.16, 2.44)			1.24	(0.89, 1.73)				
Lower extremity performance										
Intact (SPPB 10–12)			1.00		1.00					
Impaired (SPPB 0–9)			3.31	(2.62, 4.18)	2.58	(2.05, 3.25)				
Combined association										
Intact LEP with BMI										
< 23							1.15	(0.60, 2.23)	1.09	(0.56, 2.11)
23–25							1.00		1.00	
25–30							1.18	(0.66, 2.11)	1.19	(0.67, 2.12)
≥ 30							1.48	(0.77, 2.82)	1.43	(0.76, 2.71)
Impaired LEP with BMI										
< 23							3.03	(1.73, 5.31)	2.71	(1.55, 4.74)
23–25							3.14	(1.74, 5.67)	2.73	(1.53, 4.89)
25–30							2.89	(1.68, 4.97)	2.66	(1.56, 4.53)
≥ 30							3.55	(2.02, 6.27)	3.31	(1.89, 5.81)
Use of a private car										
Car driver									1.00	
Private car passenger									2.07	(1.39, 3.07)
No use a private car									2.15	(1.41, 3.28)

*n* = 823

*PR* prevalence ratio, *CI* confidence interval, *BMI* body mass index, *LEP* lower extremity performance, *SPPB* short physical performance battery

^a^Adjusting for sex, age, chronic diseases, and education

**Fig. 2:**
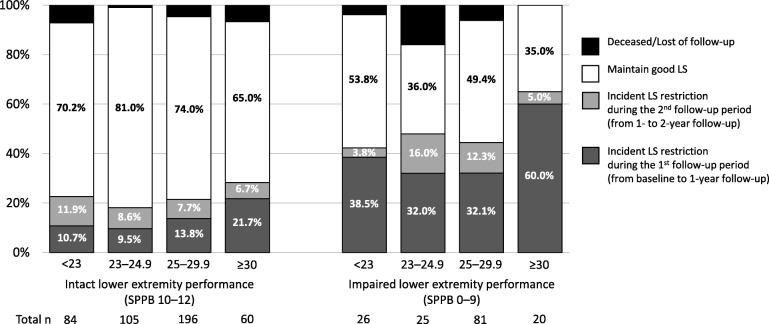
Incidence rates of life-space restriction during the 2-year follow-up according to combined lower extremity performance and BMI categories. BMI: body mass index, SPPB: Short Physical Performance Battery, LS: life-space

The flow of participants is indicated in Fig. 1. The 581 older people without life-space restriction at baseline contributed 1033 person-years during the two-year follow-up. During follow-up, incident life-space restrictions were reported by 28.3% participants (event rate 164 per 1000 person-years). Figure 2 shows the incidence rates of life-space restriction at each follow-up according to the combined BMI and SPPB categories. The incidence rate was 21.8% (18.1%–28.4% among BMI categories) among those with intact lower extremity performance and 47.4% (42.3–65.0% among BMI categories) among those with an impaired performance from baseline to two-year follow-up.

Table 3 shows the results of multiple Cox’s proportional hazard regression analyses. Subjects with a BMI ≥30.0 kg/m^2^ were 1.73 (95% CI, 1.05–2.86) times more likely to develop life-space restriction compared with people with a BMI 23.0–24.9 kg/m^2^. The corresponding figure was 2.44 (95% CI, 1.80–3.32) among people with impaired lower extremity performance vs. those with intact performance. In the model including both BMI and SPPB categories and covariates, SPPB remained significant (HR 2.39, 95% CI 1.75–3.27) but the effect of BMI was attenuated (BMI ≥30.0 kg/m^2^: HR 1.65, 95% CI 0.99–2.75). The model examining the combined effect of BMI and SPPB catergories revealed that subjects with both obesity and impaired lower extremity performance had the highest hazard for incident life-space restriction (HR 3.79, 95% CI 1.84–7.83), followed by those with impaired lower extremity performance only (HR 2.50, 95% CI 1.18–5.29), and those with obesity only (HR 1.70, 95% CI, 0.88–3.29), after adjusting for age, sex, number of chronic diseases, and year of education. In the full model including combined BMI × SPPB categories, use of a private car, and covariates, those overweight and obese subjects with impaired lower extremity performance remained significant (HR 2.77 and 3.57, respectively), and private car passengers had a significantly higher hazard for incident life-space restriction than car drivers (HR 1.96, 95% CI, 1.27–3.01). Age, numbers of chronic diseases, and years of education were statistically significant as potential confounding factors; however, sex was not.

**Table 3:** Effect of BMI and lower extremity performance on incident life-space restriction (longitudinal analysis)

	Model 1		Model 2		Model 3^a^		Model 4^a^		Model 5^a^	
	HR	95% CI	HR	95% CI	HR	95% CI	HR	95% CI	HR	95% CI
Single effect										
BMI										
< 23	1.19	(0.72, 1.97)			1.03	(0.62, 1.70)				
23–25	1.00				1.00					
25–30	1.21	(0.80, 1.84)			1.07	(0.70, 1.62)				
≥ 30	1.73	(1.05, 2.86)			1.65	(0.99, 2.75)				
Lower extremity performance										
Intact (SPPB 10–12)			1.00		1.00					
Impaired (SPPB 0–9)			2.44	(1.80, 3.32)	2.39	(1.75, 3.27)				
Combined effect										
Intact LEP with BMI										
< 23							1.02	(0.54, 1.93)	1.00	(0.53, 1.89)
23–25							1.00		1.00	
25–30							1.06	(0.61, 1.84)	1.06	(0.61, 1.83)
≥ 30							1.70	(0.88, 3.29)	1.78	(0.92, 3.47)
Impaired LEP with BMI										
< 23							2.40	(1.16, 4.97)	2.03	(0.98, 4.22)
23–25							2.50	(1.18, 5.29)	1.91	(0.90, 4.08)
25–30							2.57	(1.46, 4.52)	2.77	(1.57, 4.87)
≥ 30							3.79	(1.84, 7.83)	3.57	(1.72, 7.38)
Use of a private car										
Car driver									1.00	
Private car passenger									1.96	(1.27, 3.01)
No use a private car									1.51	(0.87, 2.62)

581 people without life-space restriction at the baseline contributed 1033 person-years during two-year follow-up

*HR* hazard ratio, *CI* confidence interval, *BMI* body mass index, *LEP* lower extremity performance, *SPPB* short physical performance battery

^a^Adjusting for sex, age, chronic diseases, and education

## Discussion

This two-year prospective study reveals that community-dwelling older people who are overweight or obese and also have impaired lower extremity performance exhibit a higher risk of both current and future life-space restriction than those who are not overweight or obese, with or without impaired lower extremity performance. Furthermore, not driving a car also renders older people vulnerable to having a restricted life-space. These results suggest that we should approach the subject of life-space mobility in older people from the aspects of both physical characteristics and transportation options.

We found that impaired lower extremity performance was a crucial contributing factor to incident life-space restriction. Previous cross-sectional studies have established that a good lower extremity performance in older adults is an important factor underlying better life-space mobility [4–6]. Lower extremity performance also contributes to incident mobility disability and limitations in ADL [27–29]. Most previous longitudinal studies have assessed the self-reported ability to perform specific tasks (e.g., walking a half mile, climbing stairs, using toilets, or bathing) with or without difficulties or help. A life-space assessment has the advantage of revealing how far participants move, which is determined by their internal physiologic capacity and their immediate environmental challenges [3]. The results of the present study suggest that lower extremity performance may correspond to the internal physiological capacity contributing to individuals’ life-space, and it may also play an important role when addressing environmental challenges.

Our results support previous cross-sectional study findings that highly obese older people (BMI ≥35.0 kg/m^2^) were significantly more likely to attain low life-space mobility scores [5]. Furthermore, systematic reviews and meta-analyses of longitudinal studies have revealed that obesity was a strong predictor of long-term risk for mobility disability and limitations in ADL [30, 31]. Excess body weight in older individuals may cause a mechanical burden potentially leading to altered walking patterns with lower energetic efficiency [32] and overall strain experienced when in motion. Additionally, increasing body weight triggers lower extremity pain (especially in the knee and ankle), which may contribute to mobility disability in older people. [30]. The joint pain may induce fear of movement and avoidance of weight-bearing tasks that trigger pain [33]. These negative consequences associated with obesity may correspond to a poor internal physiological capacity, making it difficult to tackle environmental challenges. Therefore, life-space in obese people is more likely to be restricted. After adjusting for lower extremity performance and covariates, however, the statistical significance of obesity was lost in the current study. As indicated above, BMI considerably impacts lower extremity performance and chronic diseases and may be a confounding factor itself [34].

The principal finding of the present study was that impaired lower extremity performance combined with overweight or obesity increased the risk of incident life-space restriction more than either one alone. Recently, dynapenic obesity, a condition of coexisting low muscle strength and obesity, received attention as a relative or contributing factor for falls [35], a decline in physical function [36, 37], and limitations in mobility and ADL [36]. Stenholm et al. [36] showed that obese older persons with low knee extensor muscle strength experienced significantly greater declines in walking speed and mobility than persons with either condition alone over a six-year follow-up. People with poor lower extremity muscular function may not have enough physical capacity to carry their body mass, and, consequently, their life-space mobility declines even further than those with only obesity or impaired lower extremity performance. Among people with impaired lower extremity performance, its negative impact was compensated by the use of a private car in those with low or normal weight but not in those who were overweight or obese. These results suggest that improving alternative transportation options may aid those people with low or normal weight to maintain their life-space even if their lower extremity performance is impaired. However, for obese people improvements in transportation options alone may not be sufficient to prevent life-space restriction.

The present study revealed that driving one’s private car was important for maintaining life-space in community-dwelling older people. This result is in line with previous findings showing an association between driving a car and a larger life-space [18, 19]. Shah et al. [19] reported that people who were licensed to drive were less likely to be restricted in their life-space over the average four-year follow-up compared to people without a driver’s license. The results of this study have also revealed that use of a private car, not as a driver but as a passenger, was associated with life-space restriction independently from individuals’ physical characteristics, which is consistent with a previous cross-sectional study [20]. However, the risk of incident life-space restriction was increased without statistical significance in people who did not use a private car in the present study. Even among those people, some may have frequently used public transportation modes, which are also associated with life-space [38, 39]. According to a previous cross-sectional study, the risk of life-space restriction in public transportation users did not differ significantly from private car drivers among older people without walking difficulties [20]. Further studies taking into consideration the use of public transportation modes are warranted.

The strengths of the present study were the use of high-quality longitudinal data with an excellent follow-up rate for a large population-based sample and exploring the contributing factors of life-space restriction, which is a topical issue, by focusing on both modifiable physical factors and an environmental factor. However, several study limitations warrant further consideration. Firstly, BMI was calculated from self-reported height and weight, not from objective measurements. Secondly, because of the bias of the participants’ BMI distribution, we classified those with BMI < 23.0 kg/m^2^ as low weight. However, this classification does not conform to the WHO guidelines [22] which state that a BMI < 18.5 kg/m^2^ should be classified as underweight. This change may have resulted in an underestimation of the contribution of low weight on incident life-space restriction. Thirdly, we did not collect BMI and SPPB data at the follow-ups. Therefore, we could not address whether a change in BMI or lower extremity performance in older age contributes to incident life-space restriction.

## Conclusions

Community-dwelling older adults with impaired lower extremity performance have an increased risk of both current and future life-space restriction, especially when overweight or obese. Furthermore, not driving a private car renders older people vulnerable to life-space restriction. Programs to improve lower extremity performance and to prevent excess body weight may have the potential to maintain life-space and even prevent social isolation, a potential consequence of restricted life-space mobility. Improvements in alternative transportation options, especially for older people who do not drive a car, is also essential.

## Abbreviations

ADL: Activities of daily living
BMI: Body mass index
CI: Confidence interval
HR: Hazard ratio
LISPE: Life-Space Mobility in Old Age
PR: Prevalence ratio
SPPB: Short Physical Performance Battery
